# N-Glycolylneuraminic Acid in Animal Models for Human Influenza A Virus

**DOI:** 10.3390/v13050815

**Published:** 2021-05-01

**Authors:** Cindy M. Spruit, Nikoloz Nemanichvili, Masatoshi Okamatsu, Hiromu Takematsu, Geert-Jan Boons, Robert P. de Vries

**Affiliations:** 1 Department of Chemical Biology & Drug Discovery, Utrecht Institute for Pharmaceutical Sciences, Utrecht University, 3584 CG Utrecht, The Netherlands; c.m.spruit@uu.nl (C.M.S.); g.j.p.h.boons@uu.nl (G.-J.B.); 2 Division of Pathology, Department of Biomolecular Health Sciences, Faculty of Veterinary Medicine, Utrecht University, 3584 CL Utrecht, The Netherlands; n.nemanichvili@uu.nl; 3 Laboratory of Microbiology, Department of Disease Control, Graduate School of Veterinary Medicine, Hokkaido University, Sapporo 060-0818, Hokkaido, Japan; influenza.pesti@gmail.com; 4 Department of Molecular Cell Biology, Faculty of Medical Technology, Graduate School of Health Sciences, Fujita Health University, 1-98 Dengakugakubo, Kutsukake, Toyoake 470-1192, Aichi, Japan; htakema@fujita-hu.ac.jp; 5 Complex Carbohydrate Research Center, University of Georgia, Athens, GA 30602, USA

**Keywords:** influenza, animal model, N-glycolylneuraminic acid, N-acetylneuraminic acid, CMAH, sialic acid linkage, mouse, ferret

## Abstract

The first step in influenza virus infection is the binding of hemagglutinin to sialic acid-containing glycans present on the cell surface. Over 50 different sialic acid modifications are known, of which N-acetylneuraminic acid (Neu5Ac) and N-glycolylneuraminic acid (Neu5Gc) are the two main species. Animal models with α2,6 linked Neu5Ac in the upper respiratory tract, similar to humans, are preferred to enable and mimic infection with unadapted human influenza A viruses. Animal models that are currently most often used to study human influenza are mice and ferrets. Additionally, guinea pigs, cotton rats, Syrian hamsters, tree shrews, domestic swine, and non-human primates (macaques and marmosets) are discussed. The presence of NeuGc and the distribution of sialic acid linkages in the most commonly used models is summarized and experimentally determined. We also evaluated the role of Neu5Gc in infection using Neu5Gc binding viruses and cytidine monophosphate-N-acetylneuraminic acid hydroxylase (CMAH)^−/−^ knockout mice, which lack Neu5Gc and concluded that Neu5Gc is unlikely to be a decoy receptor. This article provides a base for choosing an appropriate animal model. Although mice are one of the most favored models, they are hardly naturally susceptible to infection with human influenza viruses, possibly because they express mainly α2,3 linked sialic acids with both Neu5Ac and Neu5Gc modifications. We suggest using ferrets, which resemble humans closely in the sialic acid content, both in the linkages and the lack of Neu5Gc, lung organization, susceptibility, and disease pathogenesis.

## 1. Introduction

Infection of humans by influenza A viruses starts at the epithelium cells in the upper respiratory tract, where the hemagglutinin (HA) on the outside of a virus particle binds to glycans with a terminal sialic acid. The terminal sialic acids can be linked through an α2,3 or α2,6 bond to the penultimate galactose. In humans, mainly α2,6 linked sialic acids are present in the upper respiratory tract. The expression of α2,3 and α2,6 linked sialic acids varies between species and tissues [1].

**Figure 1:**
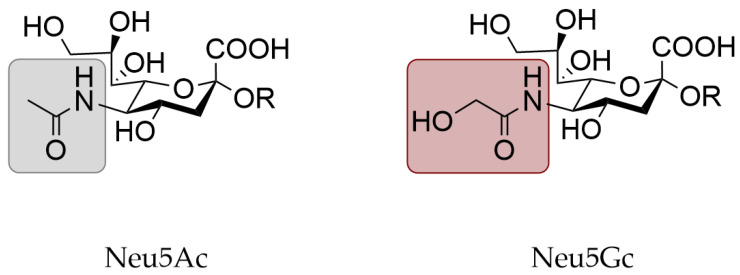
Structure of N-acetylneuraminic acid (Neu5Ac) and N-glycolylneuraminic acid (Neu5Gc).

Another variable in the influenza receptor is the type of sialic acid, of which N-acetylneuraminic acid (Neu5Ac) and N-glycolylneuraminic acid (Neu5Gc) are the main species (Figure 1). The majority of influenza A viruses use a glycan with a terminal Neu5Ac as their receptor, although some strains use Neu5Gc instead [2,3]. Importantly, only Neu5Ac is present in humans [4,5,6]. Human influenza A viruses specifically bind α2,6 linked sialic acids.

Neu5Gc can be produced in animals that express an active form of the enzyme cytidine monophosphate-N-acetylneuraminic acid hydroxylase (CMAH), which facilitates the hydroxylation of Neu5Ac to turn it into Neu5Gc. However, the gene encoding CMAH, mainly expressed in mammalian species, has been lost partially or completely in several events during evolution [4]. Possibly, the negative selection of Neu5Gc was induced by lethal pathogens binding to Neu5Gc. Therefore, the loss of Neu5Gc protected individuals from infection with these pathogens [7]. The presence of an intact CMAH gene does not automatically lead to high expression of Neu5Gc in all tissues [8]. The expression of Neu5Gc in species used as animal models for human influenza has received little attention so far and no clear overview of this expression is available.

Proper animal models are essential for fundamental and applied research on human influenza viruses, vaccines, and antivirals. Often considered factors for choosing an animal model are the experimental costs, disease pathogenesis, and susceptibility. Currently, the sialic acid linkage and especially the Neu5Gc content are often overlooked. Human influenza viruses mainly bind α2,6 linked Neu5Ac, while many animal models express Neu5Gc. The lack of correct sialic acid receptors in animal models could skew the results of a study since adaptation of a virus may be required before a successful infection is possible. The animal models mostly used to study human influenza are ferrets (*Mustela putorius furo*) and mice (*Mus musculus*). While ferrets mainly express the human receptor (α2,6 linked Neu5Ac), mice also express Neu5Gc and the sialic acid linkages in the respiratory tract differ from humans [1,6,9,10,11,12,13,14,15,16,17,18,19]. Other animal models that are discussed in this article are cotton rats (*Sigmodon* species), Syrian hamsters (*Mesocricetus auratus*), guinea pigs (*Cavia porcellus*), domestic swine (*Sus scrofa domesticus)*, macaques (*Macaca*), and marmosets (*Callitrichidae*). Of these animals, domestic swine are naturally infected by human influenza viruses [1].

In this article, we summarize the current knowledge on Neu5Gc expression in animal models for human influenza and supplement this with protein histochemistry stains on lung tissues. The role of Neu5Gc in influenza virus infection is still unclear. Furthermore, studies on Neu5Gc specific influenza viruses or Neu5Ac specific viruses in animal models that are rich in Neu5Gc are scarce. Therefore, we also studied the infection of H5N1 viruses that bind either Neu5Ac or Neu5Gc in CMAH^−/−^ knockout mice [20]. Interestingly, although the Neu5Gc specific H5N1 virus retained Neu5Ac binding, no significant difference in pathogenesis was observed between wild-type (WT) and CMAH^−/−^ mice. The Neu5Gc binding H5N1 virus was less infectious compared to the WT virus but this was not affected by the presence of Neu5Gc in mice. This article provides a base for choosing suitable animal models to study human influenza.

## 2. Materials and Methods

### 2.1. Virus and Cells

WT and mutant HA-Y161A influenza viruses A/Hong Kong/483/1997 (H5N1) were propagated in 10-day-old embryonated chicken eggs at 35 °C for 48 h. The collected allantoic fluid was stored at −80 °C. Madin-Darby canine kidney (MDCK) cells were cultured at 37 °C with 5% CO_2_ in minimum essential medium (MEM) (Nissui Pharmaceutical, Tokyo, Japan) supplemented with 10% non-immobilized fetal calf serum (FCS; SAFC Biosciences, Lenexa, KS, USA), 0.3 mg/mL L-glutamine (Wako Chemicals, Tokyo, Japan), 0.1 mg/mL streptomycin (Meiji Seika Pharma, Tokyo, Japan), 100 U/mL penicillin G (Meiji Seika Pharma, Tokyo, Japan), and 8 µg/mL gentamicin (Takata Pharmaceutical, Saitama, Japan).

### 2.2. Expression and Purification of HA

The pCD5 expression vector was used to clone HA-encoding cDNAs in frame with DNA sequences coding for a secretion signal sequence, a Twin-Strep (WSHPQFEKGGGSGGGSWSHPQFEK); IBA, Munich, Germany), a GCN4 leucine zipper trimerization domain (RMKQIEDKIEEIESKQKKIENEIARIKK) [21], and a superfolder green fluorescent protein (GFP) [22] or mOrange2 [23]. H5 HA encoding cDNAs of A/Hong Kong/483/1997 (both WT and mutant Y161A) or A/Vietnam/1203/2004 (both WT and mutant Y161A) were cloned into the pCD5 expression vector as described previously [24,25]. The HAs were expressed in HEK293S GnTI(-) cells and purified from cell culture supernatants as described earlier [24]. In short, transfection was performed using the pCD5 expression vectors and polyethyleneimine I. At 6 h after transfection, the transfection mixtures were replaced by 293 SFM II expression medium (Gibco, Gaithersburg, MD, USA) supplemented with DMSO (1.5%), 1% glutaMAX (Gibco, Gaithersburg, MD, USA), sodium bicarbonate (3.7 g/L), Primatone RL-UF (3.0 g/L), glucose (2.0 g/L), and valproic acid (0.4 g/L). The cell culture supernatants were collected at 5 to 6 days after transfection and HAs were purified using Strep-Tactin sepharose beads (IBA, Göttingen, Germany) according to the manufacturer’s instructions.

### 2.3. Protein Histochemistry

Sections of formalin-fixed, paraffin-embedded horse (*Equus ferus caballus*), guinea pig (*Cavia porcellus*), domestic swine (*Sus scrofa domesticus)*, ferret (*Mustela putorius furo*), and dog (*Canis lupus familiaris*) tissues were obtained from the Division of Pathology, Department of Biomolecular Health Sciences, Faculty of Veterinary Medicine, Utrecht University, the Netherlands. Syrian hamster (*Mesocricetus auratus*) lung tissue was a kind gift from Barry Rockx of the department of Viroscience, Erasmus Medical Center, the Netherlands. The lungs of five-week-old C57BL/6 (Japan SLC, Shizuoka, Japan) and CMAH^−/−^ knockout mice (*Mus musculus*) [20] were also fixed in formalin and embedded in paraffin. Protein histochemistry was performed as previously described [26,27]. Shortly, 4 µm tissue sections were deparaffinized and rehydrated. Antigens were retrieved by heating the slides for 10 min in 10 mM sodium citrate at pH 6.0. Subsequently, slides were treated with 1% hydrogen peroxide in MeOH for 30 min to inactivate endogenous peroxidases. The slides were blocked overnight at 4 °C with PBS supplemented with 3% BSA (*w*/*v*) (for protein stains) or carbo-free blocking solution (SP-5040-125; Vector Laboratories, Burlingame, CA, USA) (for *Sambuca nigra* agglutinin (SNA) stains). The slides were then stained using 100 µL of 5 µg/mL HAs after pre-complexing the proteins on ice for 20 min with mouse anti-streptag-HRP and goat anti-mouse IgG-HRP (626520; Thermo Fisher, Waltham, MA, USA) in PBS in respectively a 4:2:1 molar ratio. For anti-Neu5Gc stains, 1:300 diluted Anti-Neu5Gc IgY (Biolegend, San Diego, CA, USA) and 1:100 diluted Rabbit anti-chicken IgY-HRP (31401; Thermo Fisher) were used. For SNA stains, 100 µL of 5 µg/mL biotinylated SNA (B1305; Vector Laboratories, Burlingame, CA, USA) in PBS was used to stain the slides for 30 min. Afterward, the Vectastain ABC kit (PK-4000; Vector Laboratories, Burlingame, CA, USA) was applied according to the manufacturer’s protocol. After washing with PBS, slides were counterstained using hematoxylin and the HA binding was visualized using 3-amino-9-ethylcarbazole (AEC) (Sigma-Aldrich, Steinheim, Germany).

### 2.4. Hemagglutination Assay

Hemagglutination assays with pre-complexed HAs, at a starting concentration of 10 μg/mL, were performed with 0.5% canine (beagle, that lacks Neu5Gc [28]) and equine erythrocytes as described previously [24]. Shortly, precomplexation was performed with HAs, anti-streptag, and goat anti-mouse antibodies in a 4:2:1 molar ratio respectively.

### 2.5. Glycan Microarray Binding of HA

The previously presented glycan microarray [3] was used. HAs were pre-complexed with mouse anti-streptag-HRP (horseradish peroxidase) and goat anti-mouse-Alexa555 antibodies in a 4:2:1 molar ratio respectively in 50 µL PBS with 0.1% Tween-20 and incubated for 15 min on ice. The mixture was incubated on the glycan array slides for 90 min in a humidified chamber after which the slides were rinsed successively with PBS supplemented with 0.1% Tween-20, PBS, and deionized water. The slides were dried by centrifugation and immediately scanned as described previously [3]. The data (six replicates) was processed by removing the highest and lowest replicate after which the mean value and standard deviation were calculated over the four remaining replicates.

### 2.6. Virus Challenge in Mice

Five-week-old C57BL/6 (Japan SLC, Shizuoka, Japan) and CMAH^−/−^ knockout mice [20] (*n* = 20 mice/each strain) were acclimatized for one week before virus challenge. Mice were inoculated intranasally with the WT or mutant HA-Y161A A/Hong Kong/483/1997 (H5N1) viruses (*n* = 10 mice/each strain) using 10 times the 50% mouse lethal dose (MLD_50_) in 30 µL per mouse under anesthesia into C57BL/6 and *CMAH* knockout mice. The MLD_50_ was determined in WT C57BL/6 mice with WT A/Hong Kong/483/1997 (H5N1) virus. The infectivity titer (10 MLD_50_ = 10^3.0^ 50% egg infective dose) of the inoculum was adjusted with PBS. For 14 days following inoculation, mice (*n* = 5/each strain) were observed daily for body weight, clinical signs, and survival. Viruses were fully sequenced and evaluated for changes in amino acid 161 of the hemagglutinin before inoculation and at 3 days post infection (dpi).

### 2.7. Preparation of Lung Homogenates

At 3 dpi, mouse lungs were collected after euthanasia (*n* = 5/each strain). Lungs were homogenized with 2 mL of MEM supplemented with 10,000 U/mL penicillin G, 10 mg/mL streptomycin, 0.3 mg/mL gentamicin, 250 U/mL Nystatin (Sigma-Aldrich, St. Louis, MO, USA), and 0.5% bovine serum albumin fraction V (Roche, Basel, Switzerland). The homogenized lung tissue was centrifuged for 5 min at 4 °C and 8000 rpm. The supernatant was collected and stored at −80 °C until quantification of virus titers.

### 2.8. Virus Titration of Lung Homogenates

Plaque assays were performed as described previously [29]. Briefly, the mouse lung homogenates were diluted in MEM without FCS, applied in tenfold dilutions onto confluent monolayers of MDCK cells, and incubated at 35 °C with 5% CO_2_. After 1 h, the unbound virus was removed by discarding the supernatant and washing the cells with PBS. The cells were then overlaid with MEM containing 5 µg/mL acetylated trypsin (Sigma-Aldrich) and 1% Bacto Agar (Becton, Dickinson and Company, Franklin Lakes, NJ, USA). After incubation for 48 h at 35 °C, the cells were stained with 0.005% neutral red. After incubation for another 24 h at 35 °C, the number of plaques was counted. The number of plaque-forming units (PFU) was calculated as the product of the reciprocal value of the highest virus dilution and the number of plaques in the dilution.

## 3. Results

### 3.1. Animal Models for Human Influenza

The most used animal models for human influenza are mice [30,31,32,33,34], ferrets [30,31,32,34,35,36,37,38], and guinea pigs [31,34]. Other small animal models that are used are cotton rats [34,39,40,41] and Syrian hamsters [32,34], and recently, tree shrews have been suggested [42,43]. Commonly used larger animal models are domestic swine [44] and non-human primates (mainly macaques and marmosets) [34,41,45,46,47,48].

When comparing experimental costs, housing requirements, space usage, and handling conditions for small animal models, mice and cotton rats are less demanding than guinea pigs, ferrets, tree shrews, and Syrian hamsters [30,32,34,49,50,51,52]. Swine and non-human primates require more space and more complicated husbandry and handling conditions, associated with higher experimental costs compared to small animal models [34,49].

For the analysis of infection and immunological reactions, high availability of immunological reagents is beneficial, such as available for mice, cotton rats, swine, and non-human primates [30,34,53]. For ferrets, guinea pigs, and Syrian hamsters [34,51,54,55], only a few immunological reagents are available, which complicates the analysis of experiments.

### 3.2. Physical Characteristics of Animal Models

Comparable disease pathogenesis (sneezing, nasal discharge, lethargy, fever, weight loss, and viral shedding) in humans and animal models simplifies the analysis of the influenza virus infection. From all animal models, ferrets best represent the symptoms of human influenza virus infection, whereas mice, non-human primates, swine, cotton rats, and tree shrews moderately reflect the symptoms. On the other hand, guinea pigs and Syrian hamsters only reflect human symptoms to a lower extent [34,43,49,56].

The similarity in disease pathogenesis does not seem to be correlated to the resemblance between human and animal model lung structures. In the human lung, the major vessels in the lung and the bronchi are linked by extensive interlobular and intralobular connective tissue. Human lungs are most closely resembled by lungs of non-human primates in terms of structure, physiology, and mucosal immune mechanisms [57], but many (ethical) issues are associated with experiments with non-human primates. Swine and ferret lungs very closely resemble human lungs as well [36,53,56,58]. Furthermore, similarities in the lung organization are found in most mammals, among which guinea pigs, cotton rats, tree shrews, and Syrian hamsters [34,59,60,61]. However, mice lungs have different branching, organization of bronchi, and distribution of connective tissue [53,62].

To study the clearance and development of influenza virus infection, it is useful to have similar immune responses to influenza infection. The immune system of ferrets has not been studied in detail [31,54]. The immune systems of guinea pigs, Syrian hamsters, tree shrews, and swine, compared to murine species (mice and rats), are more similar to the human immune system [44,50,51,53,63,64,65]. Non-human primates have high genetic homology to humans and more than 90% amino acid similarity is found in the cytokines of humans and non-human primates [47,66,67], but differences in the sialic acid-recognizing immunoglobulin-like lectins are observed [68]. Interestingly, Neu5Gc regulates and influences several immunological pathways. Upon activation of T cells, upregulation of Neu5Ac and α2,3 linked sialic acids and downregulation of Neu5Gc and α2,6 linked sialic acids was observed [69,70]. Furthermore, T cells were more reactive to stimulation in the absence than in the presence of Neu5Gc [70,71]. Additionally, Neu5Gc suppresses the B cell responses and proliferation [20]. Therefore, the presence of Neu5Gc may be important in the comparison of the immune responses of different animal models. Apart from the influence of sialic acids on the immune system, humans can already have pre-existing immunity to a broad spectrum of influenza strains. This pre-existing immunity is often lacking in animal models, although pre-immune mice and ferret models are available [72,73,74,75,76].

The susceptibility of animal models to influenza viruses that are not adapted is an important factor for choosing an animal model, since an adapted virus may have different properties than the original virus. Swine are natural hosts of influenza viruses and are infected by the same subtypes of influenza as humans [1]. Furthermore, some non-human primates in nature are also infected with human influenza A viruses [46,77]. Additionally, ferrets, guinea pigs, cotton rats, Syrian hamsters, and tree shrews can be infected with human influenza viruses without adaptation [30,32,34,39,43,49,54,55]. When considering the two most often used small animal models for human influenza, ferrets are susceptible to influenza without adaptation, while the susceptibility of mice depends on both the influenza subtype and mice strains that are used. Efforts have been made to provide different strains of mice with a broad genetic background [78], of which some strains were shown to be more susceptible to H3N2 infection [79]. In general, DBA/2 mice seem to be more susceptible than C57BL/6 mice to human influenza infection [33,80,81,82,83,84]. Furthermore, genetic modification of mice in for example the IFITM3 [85], Mx1 [86], or Casp1 [87] genes can increase the susceptibility. Depending on the strain of influenza used in the research, it is important to choose an appropriate and susceptible murine strain that does not require adaptation of the virus.

### 3.3. Expression of N-Glycolylneuraminic Acid in Animal Models

The susceptibility of animal models is for the most part caused by the receptor specificity of the hemagglutinin and the sialic acids present at the site of infection. To mimic influenza virus infection in humans closely, similar sialic acids and sialic acid linkages in the respiratory tract of animal models are required. In humans, mainly α2,6 linked Neu5Ac is present in the upper respiratory tract, which is bound by human influenza A viruses [1]. Importantly, only Neu5Ac is present in humans, since the CMAH gene responsible for producing Neu5Gc (Figure 1) is not intact [4,5,6].

The linkage of sialic acids to the penultimate galactose residues in the respiratory tract of animal models has already been extensively reviewed by others and is summarized in Table 1. In short, tree shrews and ferrets have a human-like distribution of sialic acid linkages, with α2,6 linked sialic acids in the upper respiratory tract and α2,3 linked sialic acids in the lower respiratory tract. Swine mainly express α2,6 linked sialic acids and guinea pigs, rats, and Syrian hamsters express both α2,3 and α2,6 linked sialic acids. Non-human primates (macaque and marmoset) and mice mainly express α2,3 linked sialic acids. So far, no overview is available describing the presence of Neu5Gc in animal models for human influenza. Therefore, we summarized the presence of both intact CMAH genes and the expression of Neu5Gc (Table 1) in animal models for human influenza, since the presence of CMAH does not automatically lead to high expression of Neu5Gc [8]. For completeness, we added dogs and horses to the table, since these species highlight the differences in sialic acid expression between species, although they are hardly used as models for research on human influenza A viruses. Neu5Gc is present in all animal models except the ferret and marmoset. Information on the percentage of Neu5Gc in the respiratory tract with respect to the total sialic acid content was, to our knowledge, only available for swine and varied from 9–53%. In dogs, the presence of Neu5Gc depends on the breed and the consensus is that Neu5Gc is present in some Japanese breeds, while Neu5Gc lacks in all other breeds [88]. At the moment, it is unclear whether an intact CMAH gene is present in all dog breeds. For the other investigated species, the presence of an intact CMAH gene correlates with the expression of Neu5Gc.

**Figure 2:**
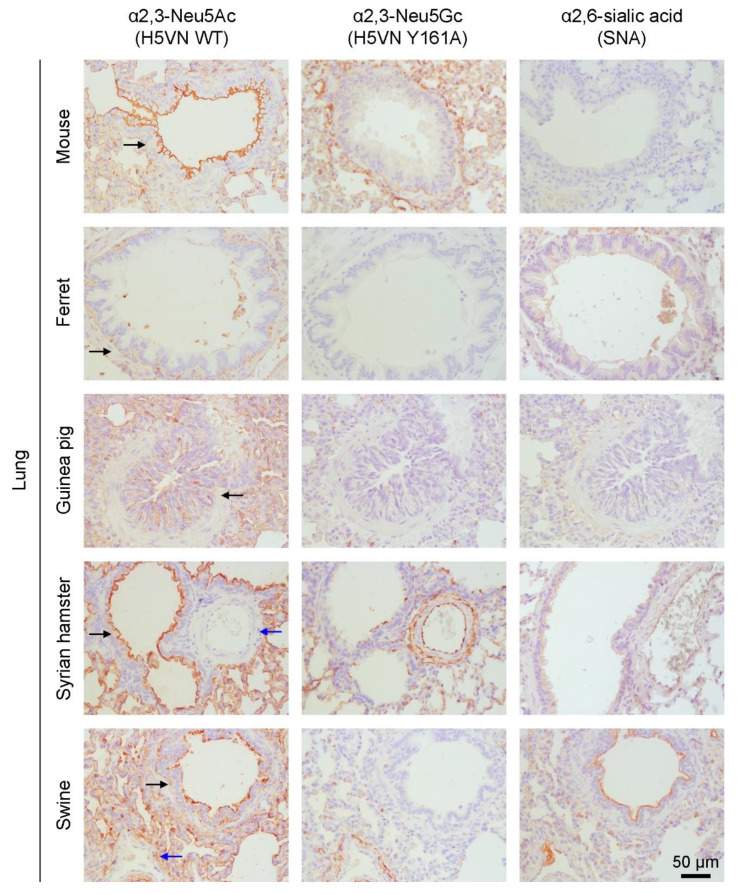
Visualization of the presence of α2,3 linked Neu5Ac (WT HA of A/Vietnam/1203/2004 (H5N1)), α2,3 linked Neu5Gc (Y161A mutant HA of A/Vietnam/1203/2004 (H5N1)), and α2,6 linked sialic acids (SNA) using AEC staining on lung tissue of mouse (C57BL/6), ferret, guinea pig, Syrian hamster, and domestic swine. Brown staining indicates binding of the HAs to the tissue and blue indicates the cells. Selected bronchioles (black arrows) and blood vessels (blue arrows) are indicated. Images are representative of two independent experiments.

The table is supplemented with protein histochemistry of the Neu5Gc content of the lungs of mice, ferrets, guinea pigs, Syrian hamsters (which was not investigated for Neu5Gc content before), and domestic swine (Figure 2). We used the WT and Y161A mutant HA of A/Vietnam/1203/2004 (H5N1) which have previously been used to specifically detect α2,3 linked Neu5Ac and α2,3 linked Neu5Gc, respectively [3]. To clarify the presence of α2,6 linked sialic acids, *Sambuca nigra* agglutinin (SNA) was used [3]. We found that Neu5Ac is expressed in all investigated lungs. In most lungs, α2,3 linked Neu5Ac was predominantly expressed in the bronchioles and alveoli, except for the ferret in which only alveolar staining was observed. Neu5Gc expression is detected throughout the tissues for all species except the ferret. Interestingly, Neu5Ac but not Neu5Gc is expressed in the bronchioles of the Syrian hamster and swine, while the inverse is true for endothelial cells in blood vessels. Thus, although Neu5Gc is present in species with an intact CMAH, expression is mainly seen in the alveoli, which are located deep in the lung. Moreover, Neu5Gc is present in the vascular system which is hardly infected by influenza A viruses. Mice hardly expressed α2,6 linked sialic acids, as expected. High expression of α2,6 linked sialic acids is observed in ferrets and swine, moderate expression in guinea pigs and Syrian hamsters. Taken together, the human respiratory tract is best resembled, in terms of sialic acids, by ferrets, which express mainly α2,6 linked Neu5Ac. On the other hand, mice mainly express α2,3 linked sialic acids, of which a part is Neu5Gc instead of Neu5Ac. We wondered if abrogating Neu5Gc from mice using CMAH^−/−^ knockout mice would make mice more susceptible to influenza A virus infection as most of those viruses exclusively bind Neu5Ac.

### 3.4. Neu5Gc as a Functional Receptor

The effect of Neu5Gc on influenza virus infection is not clear yet. Although Neu5Gc was previously suggested to act as a decoy receptor for influenza viruses [96,112], many others suggest that Neu5Gc is not a decoy receptor and Neu5Gc may even be a functional receptor. Firstly, an influenza A strain that bound Neu5Ac showed no reduced infectivity for *CMAH*-transfected cells with a higher Neu5Gc content [112]. Secondly, a mutant H3N8 virus that preferred binding to Neu5Ac over Neu5Gc only moderately infected ponies, which have a high Neu5Gc content, while another mutant virus that preferred binding to Neu5Gc infected all ponies in the experiment [111]. Thirdly, equine H7 viruses specifically bind Neu5Gc [2,3] and some other influenza A viruses bind Neu5Gc as well [2,103,113]. Some of these Neu5Gc binding viruses infect swine, with a significant amount of Neu5Gc in their respiratory tract. There is however no indication that these viruses persisted in the domestic swine population. 

**Figure 3:**
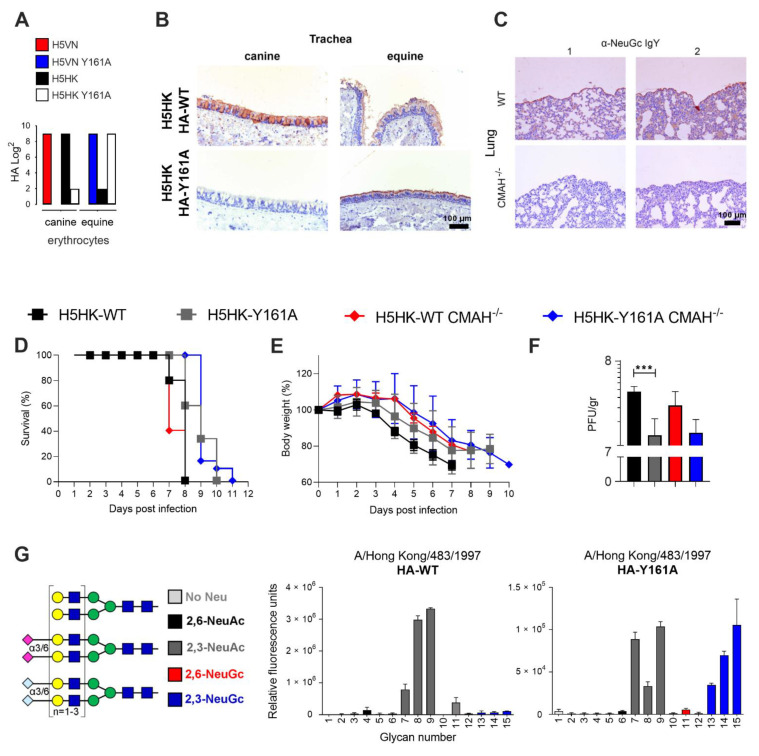
H5 WT and HA-Y161A binding properties and infectivity in WT (C57BL/6) and CMAH^−/−^ mice. (**A**) A hemagglutination assay (*n* = 3) with canine and equine erythrocytes was performed using HAs of WT and Y161A mutants of—A/Vietnam/1203/2004 (H5VN) and A/Hong Kong/483/1997 (H5HK). (**B**) Tissue binding of the HA of H5HK WT and mutant Y161A on canine and equine trachea is visualized with AEC staining (representative for three independent assays), the scale bar represents 100 µm. (**C**) Mice lungs (WT and CMAH^−/−^ knockout) were stained in duplicate with anti-Neu5Gc IgY and binding was visualized with AEC staining. WT (C57BL/6) and CMAH^−/−^ knockout mice (*n* = 5) were infected with H5HK WT and HA-Y161A mutant virus and (**D**) the survival and (**E**) body weight curves are shown, together with the (**F**) PFU per gram at 3 dpi in the lungs (*n* = 5), *** indicates a *p*-value of 0.0007 in an unpaired two-tailed t-test. (**G**) Synthetic glycans printed on the microarray (*n* = 6), either without sialic acid (structures 1–3, light gray), with α2,6-linked Neu5Ac (4–6, black), α2,3-linked Neu5Ac (7–9, dark gray), α2,6-linked Neu5Gc (10–12, red) or α2,3-linked Neu5Gc (13–15, blue). Binding specifities of WT and Y161A mutant H5HK HAs were investigated.

To investigate the effect of Neu5Gc on influenza virus infection, the CMAH^−/−^ knockout mice model [15,20,71] was used, which is not able to produce Neu5Gc and is, therefore, more similar to humans than WT mice. The CMAH^−/−^ model is also thought to produce a stronger immune response to influenza virus infection than WT mice [71]. Previously, we showed with a glycan array and hemagglutination assays that H5N1 WT virus and HA of A/Vietnam/1203/2004 (H5VN) solely bind Neu5Ac, while the Y161A mutant only binds Neu5Gc [3]. Here, we demonstrate that the WT HA of a closely related H5N1 virus, A/Hong Kong/483/1997 (H5HK), mainly binds to canine erythrocytes (Western breed), containing only Neu5Ac. The H5HK Y161A mutant mainly binds to equine erythrocytes (Figure 3A), containing primarily Neu5Gc, as indicated in Table 1. Like H5VN, the H5HK WT HA binds both equine and canine trachea, while the Y161A mutant only binds the equine trachea (Figure 3B). Therefore, the results of the hemagglutination assay and tissue staining indicated that both H5VN Y161A and H5HK Y161A are specifically binding Neu5Gc. 

To further investigate the effect of Neu5Ac and Neu5Gc binding viruses, we produced a Y161A mutant of the H5HK virus. Infection of this virus was examined in WT (C57BL/6) and CMAH^−/−^ knockout mice. Anti-Neu5Gc stains confirmed the absence of Neu5Gc in CMAH^−/−^ mice and the presence in WT mice (Figure 3C), and the presence of Neu5Ac was shown in Figure 2. Upon infection, it became clear that the WT and CMAH^−/−^ knockout mice were equally well infected, as shown by the survival curves (Figure 3D), bodyweight curves (Figure 3E), and the measurement of the PFU per gram in the lungs at three days post infection (Figure 3F). Thus, abrogating Neu5Gc did not facilitate increased virus infection and replication of a Neu5Ac binding virus and therefore it is also unlikely that Neu5Gc is used as a decoy receptor. Possibly, enough Neu5Ac was present in both the WT and CMAH^−/−^ mice to support effective infection of the Neu5Ac binding virus. The H5HK Y161A virus, binding Neu5Gc, has significantly lower pathogenicity (WT mice in Figure 3F) compared to the WT virus (*p* = 0.0007). Interestingly, this virus still infected the CMAH^−/−^ knockout mice, which was not expected based on the Neu5Gc specificity. Sequencing of the inoculated and isolated viruses (at 3 dpi) confirmed that before and after inoculation, amino acid 161 of the HA of the WT virus is a tyrosine (encoded by TAC) and this amino acid in the HA-Y161A virus is an alanine (encoded by GCC).

After this observation in vivo, we decided to do a final test on the binding properties of the WT and mutant H5HK HA on the glycan array. We found that the WT H5HK HA only binds Neu5Ac, as expected. However, the Y161A mutant binds both Neu5Ac and Neu5Gc on the glycan array (Figure 3G), whereas we expected to see only binding to Neu5Gc as with H5VN Y161A. This may explain that the H5HK Y161A virus still infected CMAH^−/−^ mice through their Neu5Ac receptors.

We now know that Neu5Gc and Neu5Ac binding on tissues and erythrocytes (chicken and horse) not always fully corresponds to glycan array data, possibly because different glycans are present in the assays [114]. We did however expect larger differences between viruses with Neu5Ac and dual Neu5Ac and Neu5Gc specificity since previously it was shown that influenza viruses with dual α2,6 and α2,3 linkage specificities transmitted less efficiently among ferrets than viruses with a specificity for a single linkage type of sialic acids [115,116,117].

## 4. Discussion

### 4.1. Relevance of Neu5Gc in Animal Models for Human Influenza

Considering space usage, ethical considerations, and experimental costs, small animal models (mouse, ferret, guinea pig, rat, tree shrew, or Syrian hamster) are preferred over non-human primates and swine for experiments with human influenza viruses. Non-human primates are also not preferred because Old World monkeys, which are mostly used for influenza research [46,47], among which the macaques, have an intact CMAH gene and express Neu5Gc [105] and mainly α2,3 linked sialic acids. They are thus very different from humans in the sialic acid content and susceptibility to human influenza A viruses. 

Guinea pigs are commonly used small animal models that express both Neu5Ac and Neu5Gc and mainly α2,3 linked sialic acids in the respiratory tract. Guinea pigs can be infected by human influenza viruses, but human symptoms of influenza infection are only reflected to a low extent and just a few immunological reagents are available. Recently, Syrian hamsters have been extensively used to investigate SARS-CoV-2 [118,119] which suggests that these animals closely resemble human respiratory tract properties. Both α2,3 and α2,6 linked sialic acids are present in their respiratory tract and Syrian hamsters can be infected by unadapted human influenza viruses. However, as we showed here for the first time, Neu5Gc is expressed in their lungs, although not on the epithelium of the bronchioles. 

In fact, we observe quite different expression profiles of Neu5Ac versus Neu5Gc not only in the Syrian hamster but also in other influenza A virus animal models. This could be the virtue of using an H5VN Y161A mutant lectin with specificity to a subset of Neu5Gc capped N-glycans to which we initially screened it for [3]. On the other hand, the commonly using anti-Neu5Gc antibody is raised against a glycolipid structure [12,120]. It would be interesting to compare several Neu5Gc specific lectins to elucidate cell- and organ-specific Neu5Gc expression profiles.

Mice are one of the most favored models because they are widely available, relatively inexpensive, and easy to keep. However, mice are hardly naturally susceptible to infection with human influenza viruses, possibly because they express mainly α2,3 linked sialic acids with both Neu5Ac and Neu5Gc modifications. Previously, mice with modified glycosylation patterns, namely ST6GAL1 knockout mice which are not able to produce α2,6 linked sialic acids, were used to investigate the infectivity of human influenza viruses. The ST6GALT1 knockout model still supported replication of human influenza viruses, although these viruses were mouse-adapted and therefore probably bound to α2,3 linked sialic acids [121]. Here, we did not observe a difference in infectivity in CMAH^−/−^ knockout and WT mice upon infection by H5N1 viruses binding Neu5Ac or with a dual Neu5Ac and Neu5Gc receptor specificity. In contrast, older literature clearly shows the importance of Neu5Gc specificity in experimental animals [111].

Although the ferret model is complicated and expensive, it is still the best model to mimic influenza infection in humans. Ferrets resemble humans very closely in terms of lung structure, susceptibility, and disease pathogenesis [34,49,54]. Importantly, the sialic acid content is very similar to that of humans, as they lack Neu5Gc and have a high α2,6 linked Neu5Ac expression in the respiratory tract. Indeed the importance of α2,6 versus α2,3 linked sialic linkage specificity has been reported extensively [122,123,124,125,126].

### 4.2. Remaining Questions on the Role of Neu5Gc and Neu5Gc Binding 

In this article, we described the presence of Neu5Gc in the animal models that are most commonly used to study human influenza. However, a quantification of the amounts of Neu5Gc present in the nasal tract, upper respiratory tract, lower respiratory tract, and lungs is still lacking. More information on this topic would provide insights into the suitability of different animal models for human influenza research. 

Since Neu5Gc is present in many animal models but is lacking in humans, it is important to investigate the role of Neu5Gc in influenza virus infection. We concluded that Neu5Gc is most likely not used as a decoy receptor. However, the exact role of Neu5Gc, and whether Neu5Gc is used as a functional receptor, remains to be elucidated. Further research comparing animal models with and without Neu5Gc and studies using Neu5Gc binding viruses would be beneficial to improve the understanding of the role of Neu5Gc in influenza virus infection.

## Figures and Tables

**Table 1:** Overview of sialic acid linkages in the respiratory tract, the presence of Neu5Gc, and the presence of an intact CMAH gene in different animal models. +: high levels, -: low levels, --: not detected.

Animal Model	Sialic Acid Linkage in the Respiratory Tract		Neu5Gc Detected	Intact CMAH Gene [4]
	α2,3	α2,6		
Mouse	+ [1]	- [1,16]	Yes [6,9,10,11,12,13,14,15,89,90] Yes (Figure 2)	Yes
Ferret	+ [17] - (upper respiratory tract) [1] + (lung) [1]	+ [17] + (upper respiratory tract) [1] - (lung) [1]	No [17,18,19] No (Figure 2)	No
Guinea pig	+ (nasal tract, trachea) [91] + (lung) [91]	+ (nasal tract, trachea) [91] - (lung) [91]	Yes [9,92] Yes (Figure 2)	Yes
Rat	+ (trachea) [93] -- (lung) [93]	+ (trachea) [93] - (lung) [93]	Yes [9,94]	Yes
Syrian hamster	+ [32]	+ [32]	Yes (Figure 2)	Yes
Tree shrew	+ [95] - (nasal tract, trachea) [43] + (lung) [43]	+ [95] + (nasal tract, trachea) [43] - (lung) [43]	Yes [95]	Yes
Swine	- [1,96] + (nasal tract) [97] - (trachea) [97] + (lung) [97,98]	+ [1,96] - (nasal tract) [97] + (trachea) [97] + (lung) [97,98]	Yes [96,98,99,100,101,102] Yes, 53% [103] Yes, 9–14% [104] Yes (Figure 2)	Yes
Marmoset	+ [45]	-- [45]	No [105]	No
Macaque	Unknown	Unknown	Yes [9,106]	Yes
Dog	+ [107]	- [3,107]	No [28,88,108] Yes [88,89,109,110]	Yes
Horse	+ [1,107,111]	-- [3,107,111]	Yes [3,8,108]	Yes
